# Assessment of *Listeria monocytogenes* virulence in the *Galleria mellonella* insect larvae model

**DOI:** 10.1371/journal.pone.0184557

**Published:** 2017-09-12

**Authors:** Mira Rakic Martinez, Martin Wiedmann, Martine Ferguson, Atin R. Datta

**Affiliations:** 1 Center for Food Safety and Applied Nutrition, Food and Drug Administration, Laurel, Maryland, United States of America; 2 Department of Food Science, Cornell University, Ithaca, New York, United States of America; University of Copenhagen, DENMARK

## Abstract

Several animal models have been used to understand the molecular basis of the pathogenicity, infectious dose and strain to strain variation of *Listeria monocytogenes*. The greater wax worm *Galleria mellonella*, as an alternative model, provides some useful advantages not available with other models and has already been described as suitable for the virulence assessment of various pathogens including *L*. *monocytogenes*. The objectives of this study are: 1) confirming the usefulness of this model with a wide panel of *Listeria* spp. including non-pathogenic *L*. *innocua*, *L*. *seeligeri*, *L*. *welshimeri* and animal pathogen *L*. *ivanovii*; 2) assessment of virulence of several isogenic in-frame deletion mutants in virulence and stress related genes of *L*. *monocytogenes* and 3) virulence assessment of paired food and clinical isolates of *L*. *monocytogenes* from 14 major listeriosis outbreaks occurred worldwide between 1980 and 2015. Larvae injected with different concentrations of *Listeria* were incubated at 37°C and monitored over seven days for time needed to kill 50% of larvae (LT_50_) and to determine change of bacterial population in *G*. *mellonella*, 2 and 24 hours post-inoculation. Non-pathogenic members of *Listeria* and *L*. *ivanovii* showed significantly (P < 0.05) higher LT_50_ (lower virulence) than the wild type *L*. *monocytogenes* strains. Isogenic mutants of *L*. *monocytogenes* with the deletions in *prfA*, *plcA*, *hly*, *actA and virR* genes, also showed significantly (P < 0.05) higher LT_50_ than the wild type strain at the inoculum of 10^6^CFU/larva. Food isolates had significantly (P < 0.05) lower virulence than the paired clinical isolates, at all three inoculum concentrations. *L*. *monocytogenes* strains related to non-invasive (gastroenteritis) outbreaks of listeriosis showed significantly (P < 0.05) lower virulence than isolates of the same serotype obtained from outbreaks with invasive symptoms. The difference, however, was dose and strain- dependent. No significant differences in virulence were observed among the serotype tested in this study.

## Introduction

Human listeriosis, caused by the pathogen *Listeria monocytogenes*, accounts for about 1600 cases and 250 deaths per year in the USA [1]. Outbreaks of listeriosis are commonly associated with severe invasive symptoms including septicemia, meningitis and spontaneous abortion among pregnant women. In general, people with compromised immune systems, neonates and the elderly are most often affected [2]. A few outbreaks with milder symptoms of febrile gastroenteritis have also been reported [3]. The non-invasive febrile gastroenteritis however has shown to have significantly higher occurrence rates and is not particularly associated with any underlying illnesses [4]. In the past, outbreaks of human listeriosis were often linked to deli meats and dairy products and most frequently involved susceptible groups including the elderly, infants, pregnant women and individuals with suppressed immune system [5]. Emergence of newer food vehicles including cantaloupe, stone fruit, caramel-coated apples and most recently, frozen foods [6–9] along with the several unusual cases of invasive listeriosis (meningitis) found among healthy children aged 5–15 years [8] emphasize the importance of a better understanding how *L*. *monocytogenes* survives and grows in fruits and vegetables and weather the different environment change virulence potential of the pathogen.

Historically, *L*. *monocytogenes* virulence assessment has been performed using laboratory animals including mice, guinea pigs and monkeys [10]. However, use of such models is frequently associated with both ethical and financial burdens. Furthermore, the number of animals needed and length of the reproduction cycle of mammals became challenging factors in research involving animals. Various invertebrate models such as *Caenorhabditis elegans (C*. *elegans)*, *Drosophila melanogaster (D*. *melanogaster)* and Zebra fish *(Danio rerio)* have also been used in the assessment of the host-pathogen interactions with *L*. *monocytogenes* [11–13]. Although some of these models have shown promise, the evaluation of the role of temperature-dependent virulence factors of *L*. *monocytogenes* is limited as many of these invertebrate models are incubated at or near room temperature. In recent years, use of larvae of the greater wax moth *Galleria mellonella* has emerged as a promising model for the assessment of virulence of numerous human pathogens including *L*. *monocytogenes* [14]. Among the various advantages of this model are low cost, easy manipulation, ethical acceptability, and the ability to incubate larvae at 37°C, the temperature of the human body and one required for the optimal expression of many key virulence factors in *L*. *monocytogenes* [15]. Most importantly, innate immune system of *G*. *mellonella* resembles to one of mammal’s, with enzymes, reactive oxygen species and antimicrobial peptides necessary to protect from bacterial infection [16].

The main objective of this study was the risk assessment of *L*. *monocytogenes* paired clinical and food isolates of *L*. *monocytogenes* associated with the major foodborne outbreaks using *G*. *mellonella* model. We also assessed the virulence potential of set of *L*. *monocytogenes* strains associated with a few self-limiting febrile gastroenteritis outbreaks. Furthermore, we aimed to demonstrate the importance of *L*. *monocytogenes* virulence factors by employing an extended panel of isogenic mutants. An understanding of the virulence potential of different *L*. *monocytogenes* strains would improve *Listeria* risk assessments and help in developing better food safety guidance as well.

## Materials and methods

### Bacterial strains and growth conditions

The different *Listeria* species, serotypes, mutations and isolates from different sources used in this study are listed in the tables. Bacteria were grown overnight at 37°C in brain heart infusion broth (BHI) (Becton Dickinson and Co., Sparks, MD) and on BHI agar plates. Bacterial cultures were washed twice and serially diluted with phosphate buffered saline (PBS). Appropriate dilutions were plated on BHI agar and incubated for 24h at 37°C for bacterial CFU count. Colony counts were used to calculated bacterial inoculum.

### *G*. *mellonella* injection and death assay

Last-instar larvae purchased from a commercial vendor (Vanderhorst, Inc., St. Marys, Ohio) were injected with bacterial inoculum in groups of 30 for each strain and for each dilution. Inoculum was administered directly to the larval hemocoel through the last left pro-leg as previously described [17]. Every trial included a group of 10 un-injected larvae as an environmental control and 10 larvae injected with PBS as a method control. Experiments were performed in at least three independent trials. Injected insects were monitored over seven days at 37°C, and the time necessary to kill 50% of larvae (LT_50_), by each inoculum was recorded. By the day seven, pupa formation was recorded in survived larvae.

### Changes in populations of *Listeria* spp. in inoculated *G*. *mellonella* larvae

To assess bacterial population change, *Galleria* larvae were infected with selected panel of different *Listeria* spp (10^6^ CFU/larva). Panel included selected non- *L*. *monocytogenes*, wild type *L*. *monocytogenes* LS1209 and isogenic mutants with the deletion in virulence and stress related genes (Tables 1 and 2). Clinical and food isolate related to the Jalisco cheese listeriosis outbreak [5] were also included. At the time points of 2 and 24 hours post-infection, respectively, five surviving larvae (approximately 1g) in each test group were randomly selected, surface sterilized with 70% ethanol, added to 1ml of PBS and crushed by vortexing. Appropriate dilutions were plated on RAPID’*L*.*mono* Medium (BIO RAD, USA) agar and incubated at 37°C for 24-36h before enumeration of the typical *L*.*monocytotgenes* colonies.

**Table 1:** LT_50_ values for non-pathogenic and pathogenic *Listeria* spp. employed in this study*.

Strain	*Listeria* spp.	LT_50_ (hours) at10^6^CFU/larva	LT_50_ (hours) at10^5^CFU/larva	Source
LS1209	*L*.*monocytogenes*	30±2	76±2	FDA
LS9	*L*. *innocua*	168±2	>168	FDA
LS166	*L*. *welshimeri*	168±3	>168	FDA
LS4	*L*. *ivanovii*	72±2	168±2	FDA
LS6	*L*. *seelingeri*	>168	>168	FDA

*Experiments were performed in three independent trials and LT_50_ values presented as a mean ± SD

**Table 2:** Change of *Listeria* population following inoculation*.

Strain	*Listeria* spp.	Change of *Listeria* population post inoculation		
		Time point 0 (CFU/larva)	2hours (CFU/larva)	24hours (CFU/larva)
LS9	*L*. *innocua*	10^6^	10^4^	10^6^
LS166	*L*. *welshimeri*	10^6^	10^4^	10^5^
LS4	*L*. *ivanovii*	10^6^	10^4^	10^7^
LS6	*L*. *seelingeri*	10^6^	10^4^	10^5^
LS1209	*L*. *monocytogenes*, 10403S, WT	10^6^	10^4^	10^8^
LS1212	*L*. *monocytogenes* 10403S *Δhly*	10^6^	10^3^	10^3^
LS1227	*L*. *monocytogenes* 10403S *ΔprfA*	10^6^	10^4^	10^6^
LS411	Jalisco cheese outbreak, food	10^6^	10^4^	10^9^
LS412	Jalisco cheese outbreak, clinical	10^6^	10^4^	10^9^

* Results represent one of two independent experiments.

### Statistical analysis

The life-table method was used to produce non-parametric estimates of the survivor functions and the homogeneity of the survival curves across strains was tested using the Wilcoxon test [18]. The analyses were stratified by dose and p-values were Bonferroni adjusted. Data was analyzed using SAS/STAT software, VERSION 9.4, (c) 2002–2012, SAS Institute Inc., Cary, NC.

## Results

### *Listeria* spp. in *G*. *mellonella* model

**Fig 1:**
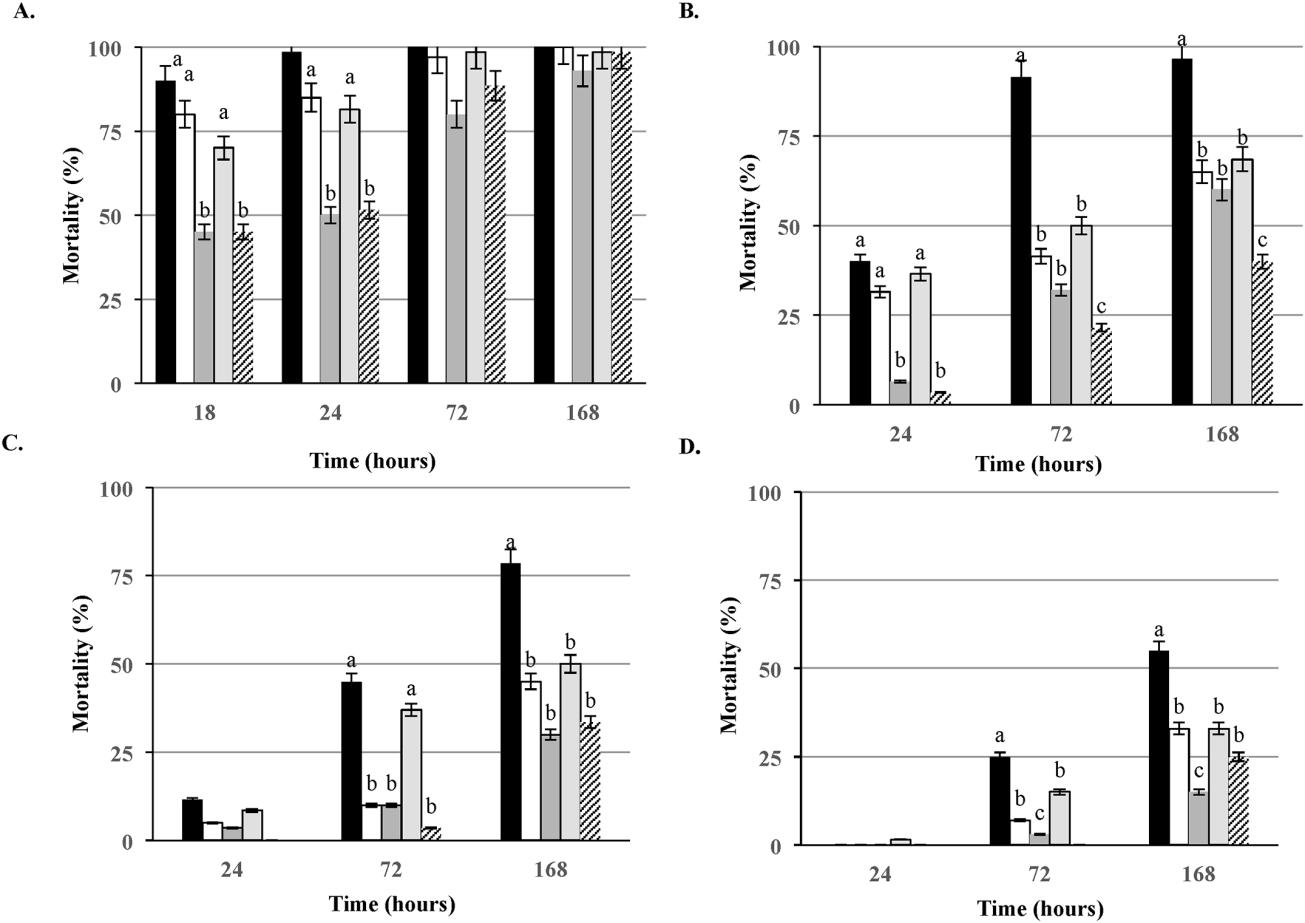
Comparison of virulence of different *Listeria* spp. in *Galleria* model. Larvae were infected with *L*. *monocytogenes* LS1209 (designated with black square) along with *L*. *innocua* LS9 (designated with white square), *L*. *welshimeri* LS166 (designated with dark gray square), *L*. *ivanovii* LS4 (designated with gray framed square) and *L*. *seeligeri* LS6 (designated with dark upward diagonal square) at the doses of (A) 10^7^ CFU/larva (B) 10^6^CFU/larva, (C) 10^5^ CFU/larva (D) 10^4^ CFU/larva. a > b >c (P<0.05) LT_50_ presents time necessary to kill 50% of larvae. Presented results are average of three independent trials.

In order to establish a range of *Listeria* concentrations for the virulence studies in *G*. *mellonella* model, we compared a few representative species of *Listeria*, including *L*. *monocytogenes* LS1209 (Table 1). The results (Fig 1) revealed similar LT_50_ (time to kill 50% of the population) values of 18–24 hours at the highest doses of 10^7^CFU/larva. The LT_50_ was significantly (P < 0.05) higher for *L*. *seeligeri* LS6 and *L*. *welshimeri* LS166, 24 hours post inoculation, while the rest of non-*L*.*monocytogenes* strains did not significantly differ from the reference *L*. *monocytogenes* strain (Fig 1A). At the inoculum of 10^6^ CFU/larva the LT_50_ for the reference *L*. *monocytogenes* LS1209, was 2 fold lesser than the *L*. *ivanovii* and 4–6 fold smaller than the rest of the non- *L*. *monocytogenes* strains tested. Significantly (P < 0.05) lower mortality was observed at 24, 72 and 168 hours (Fig 1B) for *L*. *seeligeri* LS6 and *L*. *welshimeri* LS166 compared to *L*.*monocytogenes*. Similar results were also obtained when larvae were infected with the doses of 10^5^ and 10^4^CFU, separately (Fig 1C and 1D). Among the non- *L*. *monocytogenes* strains employed in this study, *L*. *ivanovii* expressed the highest virulence potential, most significantly (P < 0.05) at the doses of 10^5^ CFU/larva after 72 h of incubation (Fig 1C). The lowest doses of 10^4^ CFU/larva produced LT_50_ of < 168 hours for *L*. *monocytogenes*, while the mortality of larvae infected with the rest of the tested strains remained well below 50% (Fig 1D). Throughout the experiment no death among environmental and method control groups have been detected.

### Change in populations of *Listeria* spp. in inoculated *G*. *mellonella* larvae

In order to monitor the fate of the injected *Listeria*, we enumerated viable *Listeria* (CFU) in the selected *Listeria* spp., 2 and 24 hours post inoculation (Table 2). Bacterial counts indicated rapid decreas of all tested strains 2 hours post inoculation followed by the increase recorded at the 24h time point. Increase, however, was strain related. Populations of LS411 and LS412, isolates from Jalisco cheese listeriosis outbreak were significantly (P<0.05) higher 24 hours post inoculation than the other tested *Listeria* strains. *L*. *monocytogenes hly* mutant at 2h was reduced by a factor of 10^3^ following the inoculation and stayed at that level even after 24h.

### *L*. *monocytogenes* isogenic mutants in *G*. *mellonella* model

**Fig 2:**
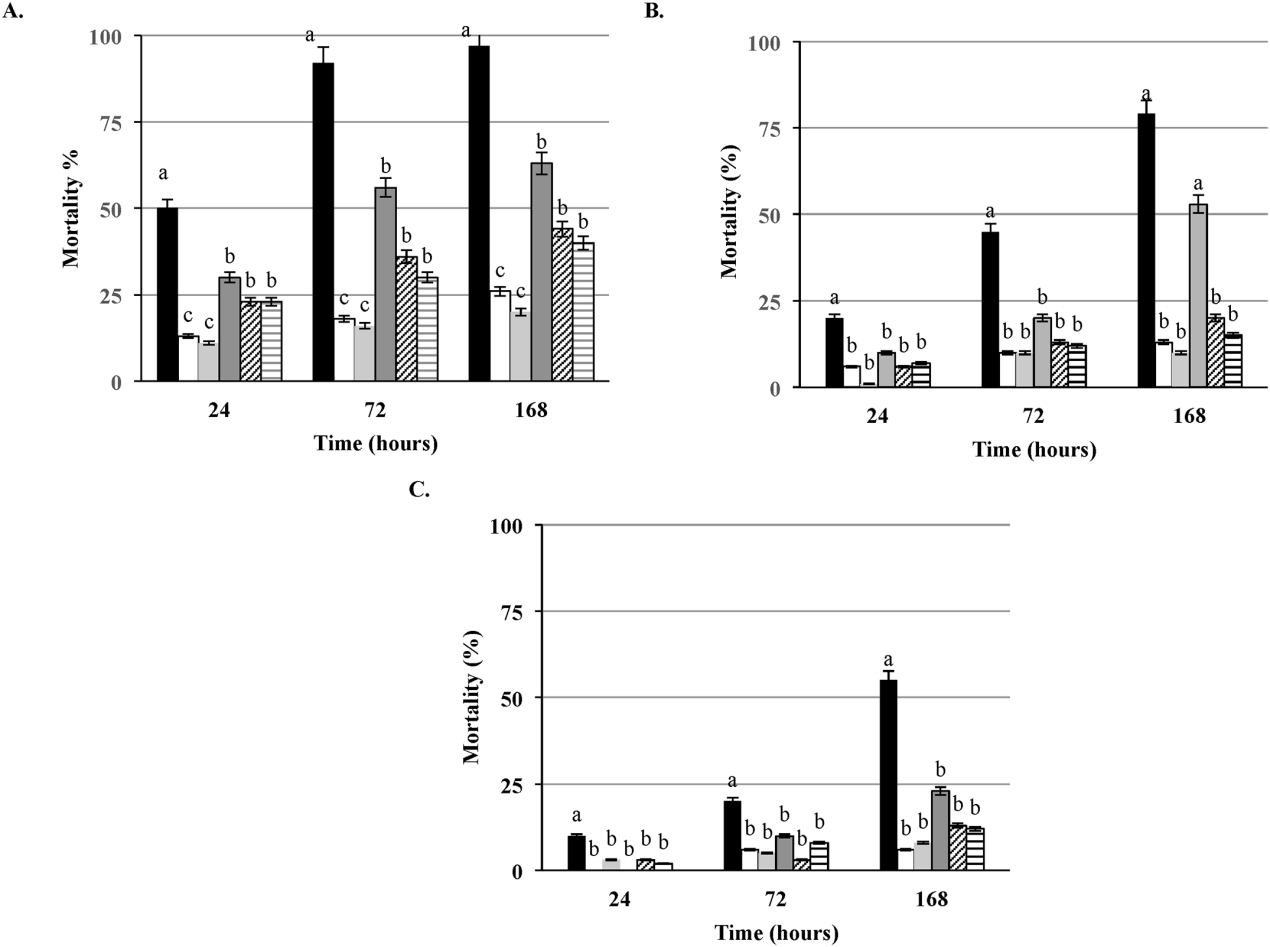
Comparison of virulence of *L*. *monocytogenes* isogenic mutants in *G*. *mellonella*. Comparison between wild type *L*. *monocytogenes* strain LS1209 (designated with black square) and isogenic mutants with deletion in *prfA* LS496 (designated with white square) *hly* LS1212 (designated with light gray square), *plcA* LS1210 (designated with dark gray square), *plcB* LS1211 (designated with dark upward diagonal square) and *actA* LS1213 (designated with dark narrow horizontal square) in larvae infected at the doses of at the doses of (A) 10^6^ CFU/larva (B) 10^5^ CFU/larva, (C) 10^4^ CFU/larva > b >c (P<0.05). Presented results are average of three independent trials.

**Fig 3:**
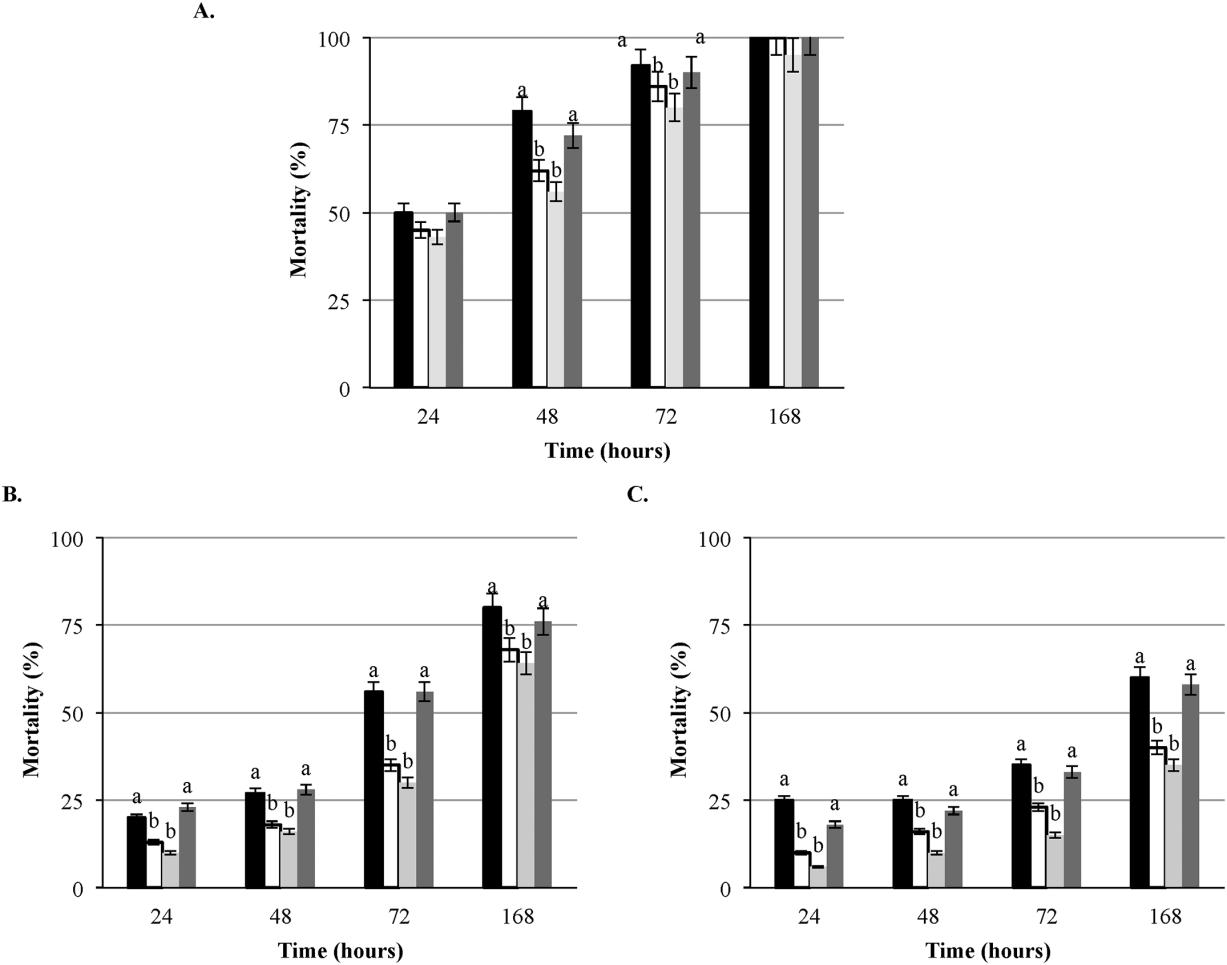
*L*. *monocytogenes* isogenic mutants with deletion in *inlA*, *inlB* and *inlAB* in *Galleria*. Mortality of larvae infected with the wild type *L*. *monocytogenes* strain LS1209 (designated with black square) and isogenic mutants with deletion in *inlB* LS1214 (designated with white square), *inlAB* LS 1215 (designated with light gray square) and *inlA* LS1222 (designated with dark gray square) at the doses of (A) 10^6^ CFU/larva (B) 10^5^ CFU/larva, (C) 10^4^ CFU/larva, a > b (P<0.05). Presented results are average of three independent trials.

A panel of 16 strains (Table 3) with the deletion in relevant virulence and oxidative stress genes along with the parental strains LS1209 and LS1223 was employed for virulence assessment in the *G*. *mellonella* model. Although deletion of the *hly* and *prfA* genes, separately, did not completely attenuate the virulence in larvae, mortality of larvae over the 168 hours of incubation was significantly (P < 0.05) lower compared to the parental strain, as well as the other isogenic mutants tested in all three doses (Fig 2A–2C). Interestingly, deletion of the *plcA* gene resulted in a lower mortality of larvae compared to the virulence of Δ*plcB*, Δ*hly*, Δ*prfA* and, *ΔactA* mutants and was dose-dependent (Fig 2A–2C). Compared to the parental strain, the virulence of Δ*plcA* was significantly (P < 0.05) lower (higher LT_50_) at each of the tested doses. Deletion of *actA* gene resulted with the significantly (P < 0.05) lower mortality of larvae compared to the wild type strain. Mutants with deletions of the *inlB*, and *inlAB* genes expressed lower killing potential than the parental strain. The difference was statistically significant (P < 0.05) only at the doses of 10^6^ and 10^5^ CFU/larva (Fig 3B and 3C). The mutant strain with the deletion of *inlA* demonstrated similar virulence to the parental strain (Fig 3A–3C).

**Table 3:** LT_50_ of *G*. *mellonella* larvae at 37°C after inoculation with *L*. *monocytogenes* isogenic mutant #.

Strain	Genotype	LT_50_ (hours) at10^6^CFU/larva	LT_50_ (hours) at10^5^CFU/larva	Source
LS1209	*L*. *monocytogenes*, 10403S, (WT*)	30±2	76±2	CU-FSL **
LS433	*L*. *monocytogenes* 10403S *ΔsigB*	30±2	80±2	CU-FSL
LS1210	*L*. *monocytogenes* 10403S *ΔplcA*	48±3	120±2	CU-FSL
LS1211	*L*. *monocytogenes* 10403S *ΔplcB*	72±3	168±3	CU-FSL
LS1212	*L*. *monocytogenes* 10403S *Δhly*	90±2	>168	CU-FSL
LS1213	*L*. *monocytogenes* 10403S *ΔactA*	64±5	144±4	CU-FSL
LS1222	*L*. *monocytogenes* 10403S Δ*inl*A	40±2	80±2	CU-FSL
LS1214	*L*. *monocytogenes* 10403S *ΔinlB*	64±2	120±2	CU-FSL
LS1215	*L*. *monocytogenes* 10403S *ΔinlAB*	64±3	124±2	CU-FSL
LS1216	*L*. *monocytogenes* 10403S *ΔinlABΔprfA*	90±2	>168	CU-FSL
LS1217	*L*. *monocytogenes* 10403S *ΔsigB* Δ*hly*BΔ*plc*B	90±2	>168	CU-FSL
LS1227	*L*. *monocytogenes* 10403S *ΔprfA*	90±2	>168	CU-FSL
LS1223	*L*. *monocytogenes* H7858 WT	24±2	64±2	CU-FSL
LS1224	*L*. *monocytogenes* H7858 *ΔvirR*	72±2	168±2	CU-FSL
LS1225	*L*. *monocytogenes* H7858 *ΔvirS*	32±3	72±3	CU-FSL
LS1226	*L*. *monocytogenes* H7858 *ΔsigBΔvirR*	72±4	168±3	CU-FSL

^#^ Experiments were performed in three independent trials and LT_50_ values presented as a mean ± SD

* Wild type strain

** Cornell University Food Safety Laboratory collection

### *L*. *monocytogenes* response regulator mutants in *G*. *mellonella* model

**Fig 4:**
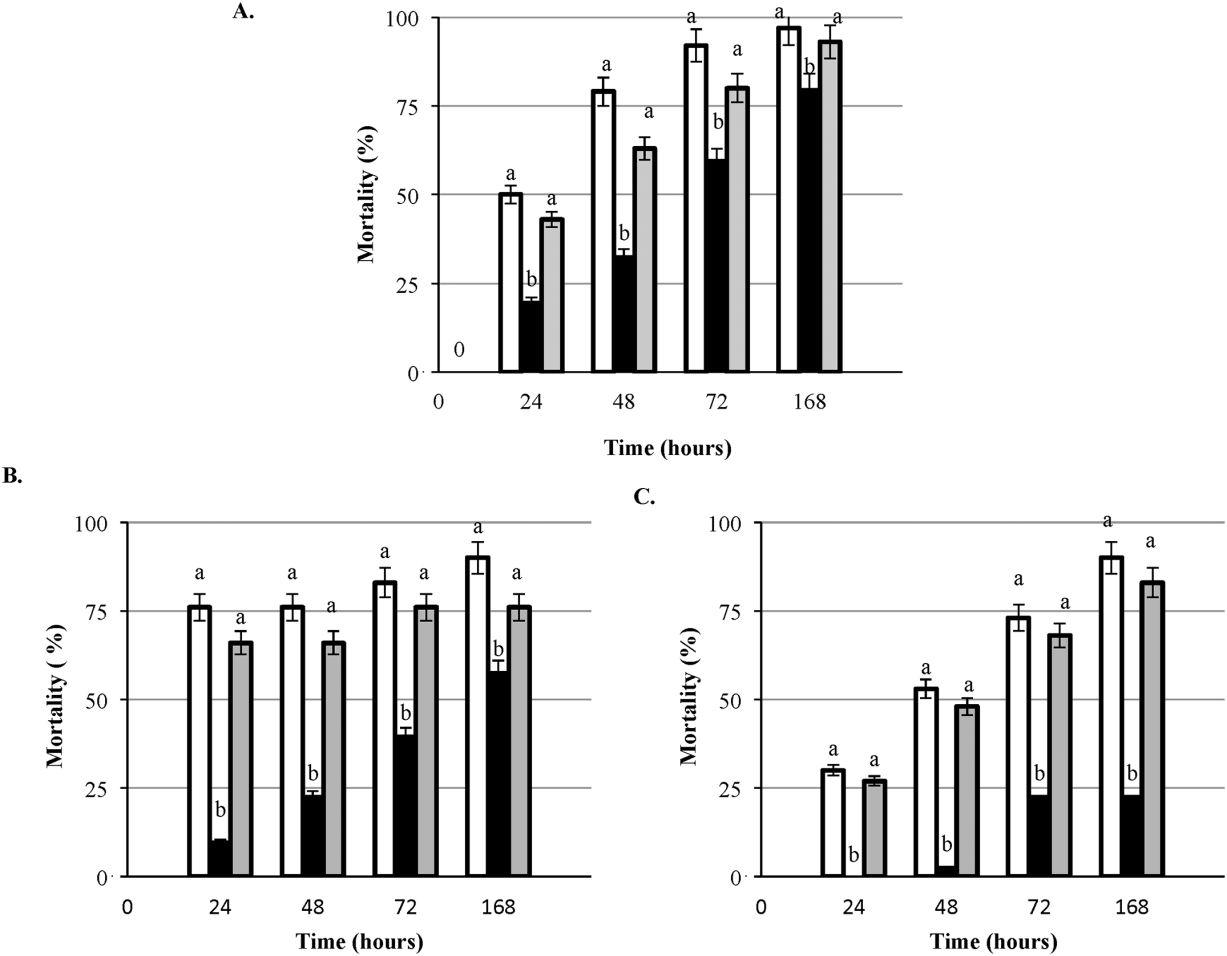
Comparison between wild type *L*. *monocytogenes* and deletion mutants in *virR* and *virS* genes in *Galleria* model. Virulence of the wild type strain LS1223 (designated with white square), *virR* LS1224 (designated with black square) and *virS* LS1225 (designated with gray square) at the doses of (A) 10^6^CFU/larva (B) 10^5^CFU/larva, (C) 10^4^CFU/larva, a > b (P< 0.05).

One of the important virulence factors in *L*. *monocytogene*s is a two-component response-regulator system termed VirR/VirS [19]. The system, encoded by *virR* and *virS* genes, regulates modifications of bacterial surface components. Isogenic deletion mutants of *virR* and *virS* in the EGD strain showed that the *virR* mutant is severely deficient in virulence while the *virS* mutation had very little effect [20]. Testing of the *L*. *monocytogenes* H7858 isogenic mutants with the deletion in *virR* and *virS* genes indicates that *virS* gene is not as essential for *L*. *monocytogenes* virulence as the presence of the *virR* gene (Fig 4). At each of the tested concentrations (Fig 4A–4C) mortality of larvae caused by the *virR* mutant was significantly (P < 0.05) lower than the larvae infected with the parental strain or of the *ΔvirS* mutant.

### Outbreak related clinical and food isolates of *L*. *monocytogenes* in the *G*. *mellonella* model

**Fig 5:**
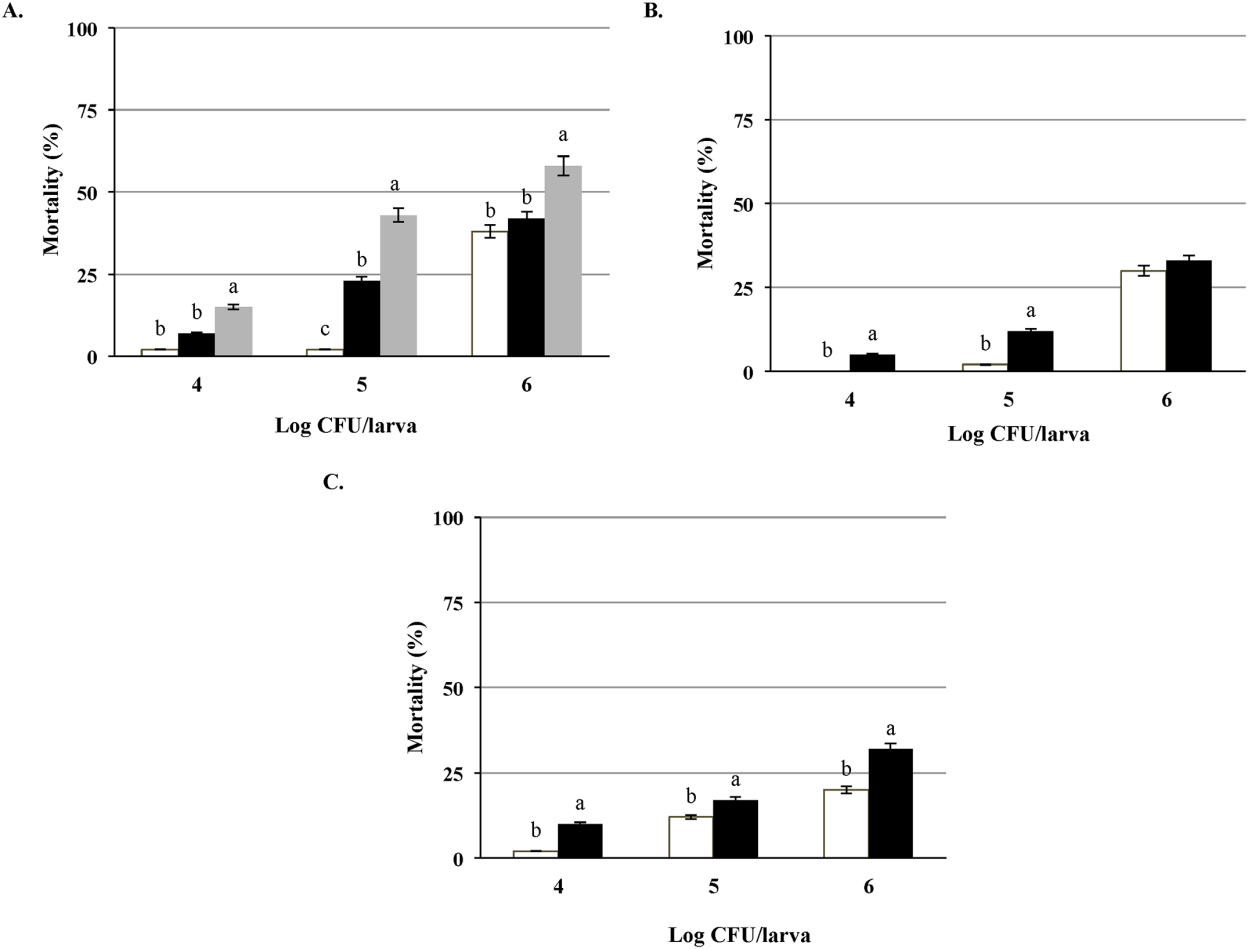
Assessment of the virulence of clinical and food isolates of *L*. *monocytogenes*. Parallel testing of the clinical isolates of *L*. *monocytogenes* (A) LS660 (designated with gray square) and food pairs isolated from chicken salad LS661 (designated with black square) and celery (□), (B) LS414 (designated with black square) and food pair isolated from coleslaw LS413 (designated with white square), (C) LS412 (designated with black square) and food pair isolated from cheese LS411 (designated with white square) at the doses of 10^4^, 10^5^ and 10^6^ CFU/larva, a > b (P<0.05).

Paired clinical and food isolates from 13 out of 14 major historical outbreaks of listeriosis (Table 4) were tested for possible differences in virulence potential. Parallel testing revealed higher virulence potential of clinical isolates of *L*. *monocytogenes* compared to their pairs recovered from food. These differences were, however, strain- and dose-dependent. The clinical isolate of *L*. *monocytogenes* strain LS620 related to celery-chicken salad outbreak [21] demonstrated significantly (P<0.05) higher virulence potential at each of the tested doses compared to the isolates obtained from the salad and celery associated with the same outbreak (Fig 5A). On the other hand, strain LS414 a clinical isolate related to the coleslaw outbreak [5] expressed significantly higher virulence potential at the lower doses of 10^5^ and 10^4^ CFU/larva, whereas doses of 10^6^ CFU/larvae were equally lethal for both the clinical and food isolate (Fig 5B). Lastly, strain LS412, a clinical isolate from the Jalisco cheese outbreak [5] resulted in significantly (P<0.05) higher mortality of larvae at the doses of 10^6^ and 10^4^CFU/larva, while difference was not significant at the doses of 10^5^CFU/larva (Fig 5C). Overall, 11 out of 13 tested clinical isolates demonstrated significantly higher virulence potential compared to their pairs isolated from the foods related to the same listeriosis outbreaks at the inoculum of 10^6^ CFU/larva. At the lower concentrations of 10^5^ and 10^4^ CFU/larva all the clinical isolates demonstrated significantly (P<0.05) higher virulence than their pairs isolated from food. We did not observe any correlation between serotypes and severity of the effect since strains LS660 (serotype 1/2a), LS411 (serotype 4b) and LS740 related to the 2011cantaloupe listeriosis outbreak [6] (serotype 1/2b); all the strains showed similar LT_50_ of 24 hours at the inoculum concentration of 10^6^CFU/larva and no significant difference in mortality of larvae.

**Table 4:** LT_50_ of *G*. *mellonella* larvae after inoculation with *L*. *monocytogenes* isolates* related to the major listeriosis outbreaks**.

Strain (origin/serotype)	Source/Illness type	LT_50_ (hours) at10^6^CFU/larva	LT_50_ (hours) at10^5^CFU/larva
LS252 (food, 1/2b)	Gastroenteritis; rice salad outbreak, Italy 1993	72±2	144±2
LS253(clinical, 1/2b)		48±3	120±2
LS402(food, 4b)	Gastroenteritis; corn salad outbreak, Italy, 1997	48±3	120±2
LS403(clinical, 4b)		40±2	90±3
LS406(stool, 4b)		72±2	120±2
LS407(stool, 4b)		72±2	144±3
LS408(stool, 4b)		64±2	144±3
LS409 (envir., 4b)		72±2	144±5
LS410 (envir., 4b)		72±2	144±2
LS404 (food, 1/2b)	Gastroenteritis; outbreak, chocolate milk, Illinois USA, 1994	72±2	120±3
LS405(clinical, 1/2b)		48±2	72±2
LS427(food, 1/2b)		72±2	120±3
LS428(clinical, 1/2b)		48±2	72±2
LS411(food, 4b)	Invasive; Jalisco cheese outbreak, California, USA 1985	48±2	130±2
LS412(clinical, 4b)		24±3	72±2
LS413(food, 4b)	Invasive; cole slaw outbreak, Halifax, Canada 1981	48±3	144±2
LS414(clinical, 4b)		40±2	72±2
LS415(food, 4b)	Invasive; pate outbreak United Kingdom, 1987–1989	32±2	64±2
LS416(clinical, 4b)		24±2	48±3
LS417(food, 4b)	Invasive; cheese outbreak Switzerland 1987,	24±2	64±3
LS418(clinical, 4b)		18±2	48±2
LS423(food, 4b)	Invasive; hot dog outbreak, USA 1998	24±4	48±2
LS424(clinical, 4b)		18±3	36±2
LS425(food, 4b)	Invasive; cheese outbreak North Carolina, USA 2000	64±2	72±2
LS426(clinical, 4b)		24±2	48±2
LS660(clinical, 1/2a)	Invasive; celery outbreak, Texas, USA, 2010	24±3	48±3
LS661(chicken salad, 1/2a)		40±2	72±2
LS662(celery, 1/2a)		40±2	120±2
LS670(food, 1/2b)	Invasive; cantaloupe outbreak, USA, 2011	48±2	144±2
LS740(clinical, 1/2b)		18±2	40±3
LS685(food, 1/2b)		28±3	48±2
LS686(food, 1/2b)		24±2	40±2
LS692 (envir., 1/2b)		40±2	72±2
LS 1044(clinical, 4b)	Invasive; caramel apples outbreak, USA, 2015	24±2	40±2
LS1046(food, 4b)		32±2	72±3

* FDA collection.

**Experiments were performed in three independent trials and LT_50_ values presented as a mean ± SD

### *L*. *monocytogenes* strains related to outbreaks of listeriosis with invasive and non-invasive (gastroenteritis) symptoms

**Fig 6:**
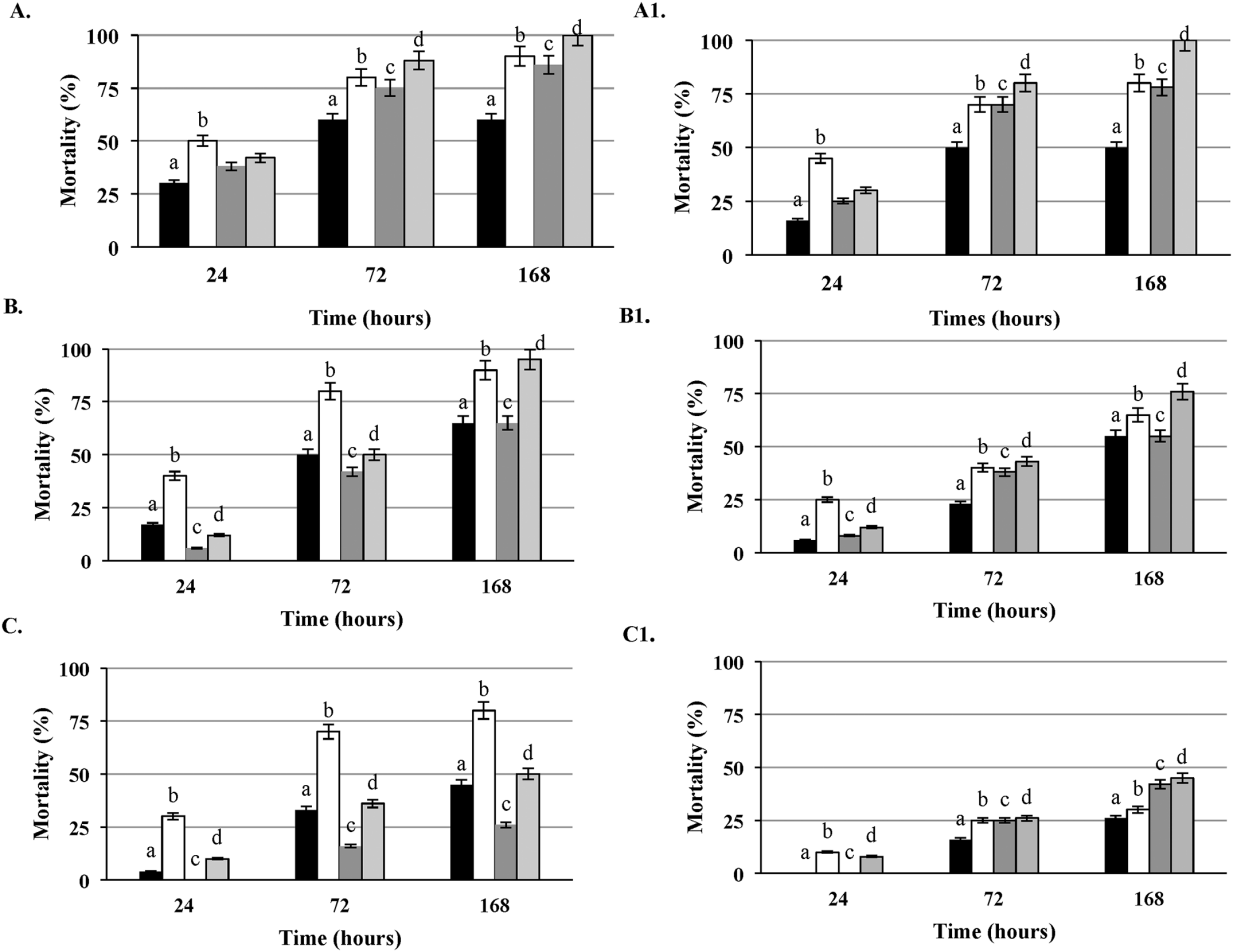
Comparison of the virulence potential of clinical and food isolates of *L*. *monocytogenes* strains related to listeriosis outbreaks with invasive and non-invasive (gastroenteritis) symptoms. Parallel testing of the clinical isolates of the same serotype related to non-invasive (LS405 –designated with black square and LS403- designated with dark gray square) and invasive (LS740-designated with white square and LS414-designated with light gray square) outbreaks at the inoculum concentrations of 10^6^CFU/larva (6A), 10^5^ CFU/larva (6B) and 10^4^ CFU/larva (6C); Paired food isolates related to non-invasive (LS404- designated with black square and LS402- designated with dark gray square) and invasive (LS670- designated with white square and LS413- designated with light gray square) outbreaks have also been compared at the inoculum concentrations of 10^6^CFU/larva (6A1), 10^5^CFU/larva (6B1) and 10^4^CFU/larva (6C1); a < b (P<0.05), c < d (P<0.05).

In order to compare the virulence of the strains related to febrile gastroenteritis and invasive outbreaks we compared isolates from three different outbreaks belonging to serotype 1/2b and 4b (Table 4). Comparison has been conducted between clinical (Fig 6A, 6B and 6C) and food isolates (Fig 6A1, 6B1 and 6C1) of same serotype related to non-invasive and invasive outbreaks at the inoculum concentrations of 10^6^CFU/larva (Fig 6A and 6A1)), 10^5^ CFU/ larva (Fig 6B and 6B1) and 10^4^ CFU/larva (Fig 6C and 6C1). Both clinical and food isolates related to invasive outbreaks showed significantly (P<0.05) higher virulence reflected as a percentage of larval mortality over the monitoring period of 168 hours. Statistical analysis were conducted relative to illness type (invasive vs non-invasive) for clinical and food isolates separately. Results from other strains listed in the Table 4 relative to illness type, not presented in Fig 6, also revealed significantly (P<0.05) higher virulence of clinical and food isolates related to invasive listeriosis outbreaks compared to the non-invasive febrile gastroenteritis outbreaks.

## Discussion

Due to the ubiquitous presence and wide distribution of *Listeria* spp. in the environment, assessment of their pathogenicity and virulence potential is of high importance. As the only food-borne human pathogen among *Listeria* spp., *L*. *monocytogenes* is of particular interest. Extensive research into human infectious diseases often requires the use of various animal species as experimental models. The most common mammalian models used are rodents, specifically mice [22]. Mice are used because of their easy availability, relatively easy manipulation, shorter life cycle and high reproductive rates. Furthermore, employment of animal models allows control of conditions and variables associated with the experiment as well as use of large number of animals necessary for valid statistical analysis. However, in recent years, use of mammalian animal models has become increasingly less acceptable. This led to the introduction of various alternative insect and nematode models [11, 23–25]. These organisms developed a very effective immune system whose function relies on humoral and cellular innate mechanisms–mimicking human host. Both, the nematode *C*. *elegans* [11] and the fruit fly, *D*. *melanogaster* [26] offer several advantages, but the inability of *C*. *elegans* worms to incubate at 37°C and lack of colonization and dissemination [27] and self-fertilization and narrow choice of infection routes in *D*. *melanogaster* [28] make them less suitable models for the study of *Listeria* spp.

Invertebrate infection model of greater wax worm *G*. *mellonella* has been employed in virulence and pathogenicity studies of various microbes including *L*. *monocytogenes* [16, 17]. Comparative analysis of various *Listeria* spp. and serotypes are also available [16]. However, the virulence assessment of different *L*. *monocytogenes* clinical and food /environmental isolates has not been previously reported. In this study we demonstrate different pathogenic potentials of clinical and food isolates related to the major historic outbreaks of listeriosis. Additionally, we compare isolates from the listeriosis outbreaks with systematic (invasive) symptoms with the isolates related to the listeriosis outbreaks with only febrile-gastroenteritis symptoms. Finally, we report the difference in pathogenicity of extended panel of *L*. *monocytogenes* isolates with single or multiple mutations in the virulence and stress related genes.

Several previous studies indicated variability in virulence among *L*. *monocytogenes* strains [29, 30]. The advantage of the employment of *G*. *mellonella* as a model for the assessment of *L*. *monocytogenes* virulence is that a large number of strains can be tested at the different infectious doses in a relatively short time and at low cost. These tests are necessary in order to better understand mechanisms of virulence and the pathogen-host immune response. Monitoring of the populations of *Listeria* spp., in the insect host, 2 and 24 hours post inoculation revealed different growth potentials among tested strains. Fast decrease in population, recorded by CFU count 2 hours post inoculation, indicates effective immune response of the insect as previously reported by Mukherjee et al. [16]. However, most of the tested strains recovered and grew thereafter (Table 2), with the exception of *L*. *monocytogenes hly* mutant. Our results demonstrated that the level of growth of pathogen inside the host does not necessarily correlate with the virulence (LT_50_).

The evaluation of *L*. *monocytogenes* strains isolated from food (n = 15), patients diagnosed with listeriosis (n = 13) and the environment (n = 6) demonstrated strong strain and dose dependent virulence potential of the pathogen. These results are also in agreement with available reports [16, 17, 31]. The novelty of this study, however, is that our data indicates higher virulence capacity of the clinical *L*. *monocytogenes* isolates when compared with their pairs isolated from foods related to the same outbreaks of listeriosis. Such difference could be attributable to epigenetic differences in the isolates originating from different sources as previously suggested [32, 33]. These authors found SNPs identified in human isolates diverge less than those from food/environmental isolates. They speculate that regulated in-host environment provides less survival pressure for pathogens compared to the environmental conditions. In our study, differences in virulence capacity were confirmed by higher larval death and a shorter LT_50_ caused by clinical isolates compared to the food isolates. The mortality and morbidity outcome of listeriosis outbreaks are complicated and depend on a host of factors including amount of pathogen ingested, food type and immune function of the consumers. Another important factor could be genotype and phenotype of the organism. A simple screening of virulence potential of *L*. *monocytogenes* strains involved in different outbreaks may provide some useful data for correlation between the severity of the disease and their virulence potential. If this is true, such information could be useful in refining Listeria risk assessment and may provide additional means of controlling human listeriosis.

Human listeriosis in healthy populations may results in self-limited gastroenteritis or can be asymptomatic; therefore such cases are rarely recorded. Several outbreaks of febrile gastroenteritis caused by *L*. *monocytogenes* have been reported [3]. The non-invasive outbreaks are often associated with high attack rates (50–80%) without any reported hospitalization and/or death. Among the tested *L*. *monocytogenes* strains three food-clinical pairs were from non-invasive febrile gastroenteritis listeriosis outbreaks [3]. Although such cases are not reported these three outbreaks had significant number of affected individuals and were linked to single food sources. Parallel testing of isolates related to the non-invasive and invasive listeriosis outbreaks revealed higher virulence potential of isolates belonging to the same serotypes and from the latter outbreak. The difference is consistent regardless of source of the tested isolates (food or clinical). Strains related to the non-invasive cases of listeriosis (with the symptoms of gastroenteritis) showed significantly (p< 0.05) lower LT_50_ when compared with isolates related to the invasive cases with severe listeriosis symptoms. The extent of difference was, however, dose and strain dependent. Laksanalamai et al. [34] compared the strains with a pan-genomic microarray and showed significance difference in genotype and transcriptome profiles between invasive and non-invasive strains. Our results with *Galleria* model showed that the non-invasive strains are also less virulent than the invasive strains. Similar results have also been reported by Franciosa *et al*., 2001 [35], suggesting the differences in DNA sequences as a possible cause of such occurrence. Previous studies with a custom designed microarray also indicated that there are differences in gene contents between the isolates from non-invasive and invasive listeriosis outbreak strains [34]. Further study is needed to understand if any one or more of these genes or difference in transcription or both are responsible for the difference in virulence observed in the present study. Although majority of the known listeriosis outbreaks had been associated with the serotype 4b strains, we did not observe any significant serotype related differences.

The genetic basis of *L*. *monocytogenes* pathogenesis is well characterized [36]. Among the genes identified, *prf*A plays a significant role in controlling *L*. *monocytogenes* pathogenicity [37]. The *prf*A gene not only controls transcription of a set of virulence related genes it also controls stress related genes and genes may not be directly associated with the pathogenicity [38]. Along with the emergence of *L*. *monocytogenes* as a major food-borne pathogen, molecular mechanisms associated with pathogen’s virulence have been extensively investigated. Creation of the isogenic mutants with deletion in the major virulence or environmental stress genes proved to be a useful tool in the assessment of *L*. *monocytogenes* pathogenicity. We used a panel of 16 wild type and mutant strains which included in-frame deletions in *prfA*, *hly*, *actA*, *plcA*, *plcB*, *inlA*, *inlB*, *inlAB*, *virR*, *virS*, and *sigB* genes. Although all of these mutants have been studied in some animal models and in-vitro tissue culture assays, to our knowledge, this is the most complete isogenic mutant panel tested in the *G*.*mellonella* model reported so far. A significantly (p< 0.05) higher virulence potential of the parent *L*. *monocytogenes* strain compared to isogenic mutants with the deletions in *prfA*, *plcA*, *plcB*, *hly*, *actA* genes, was observed in this study. Deletion of the *actA* gene in *L*. *monocytogenes* resulted with the significant decrease of larval mortality. This does not come as a surprise knowing the importance of actin based motility for the infection in vertebrate cells. Mukherjee et al. [16] previously detected intracellular rapid movement of bacteria or intracellular presence of actin “cloud”, which indicate similarity of the infection development in *Galleria* hemocytes and vertebrate cells. The significant decrease in the larval mortality caused by the absence of these five key well characterized virulence factors in *L*. *monocytogenes* confirmed *G*. *mellonella* as a useful animal model for the assessment of pathogenicity and virulence of *L*. *monocytogenes*. Thus the *Galleria* model may be useful in identifying newer virulence related genes of this foodborne pathogen.

The *vir*RS system, one of the 15 confirmed two-component systems harbored by *L*. *monocytogenes*, has important role in the control of the cell envelope stress response [20]. This two-component system is comprised of a set of genes controlled by the *vir*R regulator and the putative cognate histidine kinase of VirR known as VirS. In this study we compared isogenic mutants with the deletion of the *virR* and *virS* in *Galleria*. Our results indicate that the *virS* gene may not be significant for *L*. *monocytogenes* virulence since mortality of larvae (LT_50_) inoculated with *ΔvirS* did not differ from the parental strain. This finding is in agreement with a previous report by Mandin et al. [20] who found no difference in LD_50_ between *ΔvirS* mutant, inactivated by deleting 200 bp in the middle of the gene, and the parent *L*. *monocytogenes* strain EGD, while the LD_50_ of *ΔvirR* mutant was much higher (10^5^ CFU) compared to EGD (10^4^ CFU). In our study, deletion of the *virR* gene resulted in a significantly (p< 0.05) lower mortality of *Galleria* when compared to the wild type *L*. *monocytogenes* and *virS* mutant. The role of *virR* in antimicrobials and organic acid salts resistance has already been described [19]. These same authors also reported various antimicrobial activities of *G*. *mellonella* induced by the antimicrobial hemolymph proteins. We speculate that deletion of *virR* gene led to the stimulation of immune response of *Galleria* larvae leading to higher LT_50_ (lower virulence). Absence of *virS*, however, could have been compensated by the presence and activity of other histidine kinases or other activation mechanisms [20].

The alternative sigma factor, *sigB*, through regulation of host genes, enables survival of *L*. *monocytogenes* under challenging environmental conditions and also controls to the transcription of the gene encoding virulence regulator prfA [39, 40]. In this study, we investigated the effect of *sigB* deletion on pathogenic potential of *L*. *monocytogenes*. The results demonstrated that pathogenicity of Δ*sigB* mutant had not significantly differed from that of the parental *L*. *monocytogenes* strain. This could be due to the direct inoculation into the hemocoel, hence avoiding stressful conditions during the gastrointestinal passage. Joyce et al. 2010 [17] also reported similar reduction of *Galleria* haemocytes 24 hours after infection with wild type *L*. *monocytogenes* and Δ*sigB* mutant.

*L*.*monocytogenes* harbors a host of internalin genes of which InlA and InlB play a significant role in the invasion of the targeted cells by *L*. *monocytogenes* [41]. Testing of the *L*. *monocytogenes* mutants with deletions in *inlA*, *inlB* and *inlAB* revealed no significant difference in the virulence potential between these mutants and the wild type parent strain at any of the tested doses. In fact, *inlB* and *inlAB* mutants showed higher LT_50_ compared to the wild type strain and *inlA* mutant, with a statistically significant (p< 0.05) difference at the infection doses of 10^5^ and 10^4^ CFU/larva. In the absence of a genome sequence for *Galleria* and lack of better understanding of the host immune response, we can only speculate about the possible reasons for these effects. Direct injection of bacterial inoculum into the larval hemocoel may bypass the need for InlA function as previously observed in mice after intravenous infection [16]. Unlike the mouse model, where both *inlA* and *inlB* mutants did not show any pathogenic potential, only *inB* mutant revealed lower pathogenicity in *Galleria*. We are of the opinion that this could be host and cell-specific property of *L*. *monocytogenes*. This could be also due to some other function/s of InlB that is not relevant in mouse model of infection.

Our results confirmed dose dependent killing of *G*. *mellonella* larvae with LT_50_ of 24 hours or less at the highest dose of 10^7^ CFU/larva. These results however applied to both non- *L*. *monocytogenes* strains as well as *L*. *monocytogenes* and are in agreement with the previous findings [16, 17]. These could be due to some non-specific toxicity related to overloading the immune system with such a massive dose. Lower doses, however, demonstrated significant difference in virulence between pathogenic and non-pathogenic *Listeria* spp. This is expected as only *L*. *monocytogenes* and *L*. *ivanovii* are known to contain all the known virulence related genes, and both of them are considered to be animal pathogens with the exception that *L*. *ivanovii* has rarely caused human infection [42]. Our results thus support the previous findings that the *Galleria* model can be a suitable alternative model for *L*. *monocytogenes* infection assessment. Thus, it appears that this model may be very suitable to screen other *Listeria* species identified [16] from different environmental sources which would lead to increased understanding about the role of these species in human and animal listeriosis.
